# Are Nonnutritive Sweeteners Obesogenic? Associations between Diet, Faecal Microbiota, and Short-Chain Fatty Acids in Morbidly Obese Subjects

**DOI:** 10.1155/2019/4608315

**Published:** 2019-10-01

**Authors:** Per G. Farup, Stian Lydersen, Jørgen Valeur

**Affiliations:** ^1^Department of Research, Innlandet Hospital Trust, PB 104, N-2381 Brumunddal, Norway; ^2^Unit for Applied Clinical Research, Department of Clinical and Molecular Medicine, Faculty of Medicine and Health Sciences, Norwegian University of Science and Technology, Box 8905, N-7491 Trondheim, Norway; ^3^Regional Centre for Child and Youth Mental Health and Child Welfare, Department of Mental Health, Faculty of Medicine and Health Sciences, Norwegian University of Science and Technology, Box 8905, N-7491 Trondheim, Norway; ^4^Unger-Vetlesen Institute, Lovisenberg Diaconal Hospital, N-0440 Oslo, Norway

## Abstract

Obesity has been associated with changes in the gut microbiota and its metabolites. The study explored changes in the faecal microbiota and short-chain fatty acids (SCFA) associated with the diet (including nonnutritive sweeteners (NNSs)) and evaluated metabolic consequences in subjects with morbid obesity. The diet was assessed with a validated food frequency questionnaire. One unit of NNSs was 100 mL beverage with NNSs or 2 tablets/teaspoons of NNSs. The faecal microbiota was assessed with GA-map® dysbiosis test and SCFA with gas chromatography and flame ionisation detection. Fourteen men and 75 women with a mean age of 44.6 (SD 8.7) years, BMI 41.8 (SD 3.6) kg/m^2^, and intake of NNSs 7.5 units/day (SD 3.2; range 0–43) were included. Faecal butyric acid was positively and negatively associated with the intake of starch (partial correlation = 0.264; *p*=0.015) and NNSs (partial correlation = −0.274; *p*=0.011), respectively. NNSs were associated with changes in four out of 39 bacterial groups. Butyric acid has antiobesogenic effects, reduces insulin resistance, and improves dyslipidaemia. Since the weight-reducing effect of NNSs on obese adults trying to lose weight is dubious, it seems imprudent to use NNSs that might counteract the favourable effects of butyric acid.

## 1. Introduction

Obesity, which has nearly tripled worldwide since 1975, has health-related consequences such as increased risk of cardiovascular diseases, metabolic syndrome with diabetes type 2, musculoskeletal disorders, and cancer [1–3]. The high and increasing prevalence of obesity, estimated to 13% of the world population and mentioned as the global obesity epidemic [4], has been linked to alterations in the diet with increased intake of high fat, energy-dense food, and reduced physical activity [1]. The dietary alterations affect the gut microbiota (induce dysbiosis) and the microbiota's metabolites (e.g., straight and branched short-chain fatty acids, referred to as short-chain fatty acids (SCFA) in this paper) [5]. Evidence indicates associations between alterations of the gut microbiota and their metabolites and obesity [6–9]. Propionic and butyric acids have been ascribed antiobesogenic effects, and a high *Firmicutes/Bacteroidetes* ratio has been associated with obesity [10–16]. Although these changes have been observed in both animals and humans, a causal role of the gut microbiome in the pathogenesis of obesity has not yet been proven in humans [6, 17].

Dietary factors like fibre and starch, intake of nonnutritive sweeteners (NNSs), and use of drugs like metformin alter the gut microbiome and their metabolic products [5, 18–26]. This study aimed to explore associations between the diet and drugs, and changes in the gut microbiota and SCFA in subjects with morbid obesity, and to evaluate the metabolic consequences. Because our previous research has indicated unfavourable effects of NNSs, the study focused on the effects of NNSs [25, 27]. A secondary aim was to study direct and indirect effects (mediated via the faecal microbiota) of NNSs on SCFA.

## 2. Materials and Methods

### 2.1. Study Design

The design was a cross-sectional study in subjects with morbid obesity (MO) referred for evaluation of bariatric surgery. Results from this cross-sectional study have been reported in previous papers, and this study followed the methods of Farup and Valeur [28].

### 2.2. Participants

Consecutive subjects aged 18–65 years with MO (defined as BMI >40 or >35 kg/m^2^ with obesity-related comorbidity), referred to the Unit for Obesity at Innlandet Hospital Trust–Gjøvik, Norway, in the period from December 2012 to September 2014 were informed about the study and were asked to participate. Exclusion criteria were organic gastrointestinal disorders, major psychiatric disorders, severe somatic disorders not related to obesity, alcohol or drug addiction, and previous obesity surgery or other major abdominal surgery.

### 2.3. Accomplishment

In all participants, a medical history was taken, a physical examination was performed, and blood and faecal samples were collected. The doctors, the study nurse, and the participants filled in paper-based questionnaires. Supplementary examinations were performed at the doctors' discretion.

### 2.4. Variables

#### 2.4.1. Participants' Characteristics

The following variables were registered:

- Gender, age (years), height (m), weight (kg), BMI (kg/m^2^), coffee (cups/day), smoking (daily, previously, and never), and previous and present diseases.
- Use of metformin and other drugs (yes/no).
- The diet was assessed with a food frequency questionnaire based on the official Norwegian food composition table and validated by the University of Oslo [29]. The amount of NNSs was calculated. One unit of NNSs was defined as 100 mL NNS-containing beverage or two NNS tablets/teaspoons for use in tea or coffee.

#### 2.4.2. Faecal Samples

The producer of the microbial test, Genetic Analysis AS, Oslo, Norway, provided kits for collecting the faecal specimens. The participants collected faecal material in the kits at home and stored it at room temperature for a maximum of five days before bringing the specimen to the hospital where it was kept at minus 80°C until it was analysed [30].

*(1) Faecal Microbiota*. GA-map® dysbiosis test (Genetic Analysis AS, Oslo, Norway) was used for the analyses of the faecal microbiota [30]. The test is CE marked and has a US (Patent No. 9243297) and a European patent (Patent No. 2652145) for its technology [31]. The test is based on advances in DNA profiling using probes targeting variable regions (V3 to V7) of the bacterial 16S rRNA gene to characterise and identify bacteria present at different taxonomic levels.

The overall result is given as the Dysbiosis Index (DI) with scores 1 to 5; values above 2 indicate a microbiota profile that differs from the producer's reference population (i.e., dysbiosis). The test also reports the relative abundance of 39 bacteria at different taxonomic levels compared with a reference population (score −3 to 3) of Actinobacteria, Actinomycetales, *Bifidobacterium* spp., *Alistipes*, *Alistipes onderdonkii*, *Bacteroides fragilis*, *Bacteroides* spp. and *Prevotella* spp., *Bacteroides stercoris*, *Bacteroides zoogleoformans*, *Parabacteroides johnsonii*, *Parabacteroides* spp., Firmicutes, Bacilli, *Catenibacterium mitsuoka*, Clostridi a, *Clostridium* sp., *Dialister invisus*, *Dialister invisus* and *Megasphaera micronuciformis*, *Dorea* spp., *Eubacterium biforme*, *Eubacterium hallii*, *Eubacterium rectale*, *Eubacterium siraeum*, *Faecalibacterium prausnitzii*, Lachnospiraceae, *Lactobacillus ruminis* and *Pediococcus acidilactic*, *Lactobacillus* spp., *Phascolarctobacterium* sp., *Ruminococcus albus* and *R. bromii*, *Ruminococcus gnavus*, *Streptococcus agalactiae and Eubacterium rectale*, *Streptococcus salivarius* ssp. *thermophiles* and *S. sanguinis*, *Streptococcus salivarius* ssp. *Thermophilus*, *Streptococcus* spp., *Veillonella* spp., Proteobacteria, *Shigella* spp. and *Escherichia* spp., *Mycoplasma hominis*, and *Akkermanasia muciniphilia*. The dysbiosis scores are the producer's commercial secret.

An Alternative dysbiosis index (ADI) (scores −14 to 14), which we have claimed to separate favourable dysbiosis (positive scores) from unfavourable dysbiosis (negative scores), was also calculated [25]. The ADI is based on the relative abundance of the bacteria Alistipes, Proteobacteria and *Shigella* spp. and *Escherichia* spp., and the relative scarcity of *Bacteroides fragilis*, *Ruminococcus gnavus*, *Bacteroides* spp. and *Prevotella* spp., and *Dialister invisus.*

*(2) Faecal Short-Chain Fatty Acids*. 0.5 g of the faecal samples and distilled water containing 3 mmol/L of 2-ethylbutyric acid (as internal standard) and 0.5 mmol/L of H_2_SO_4_ were homogenized. 2.5 mL of the homogenate was vacuum distilled according to the method of Zijlstra et al. and modified by Høverstad et al. [32, 33]. The distillate was analysed with gas chromatography (Agilent 7890 A; Agilent, CA, USA) using a capillary column (serial no. USE400345H, Agilent J&W GC columns; Agilent, CA, USA) and quantified while using internal standardisation. Flame ionisation detection was employed. The total amount of all SCFA and the amount of acetic, propionic, butyric, i-butyric, valeric, i-valeric, caproic, and i-caproic acids expressed in mmol/kg wet weight were measured, and two indices were calculated:

- Index A (saccharolytic fermentation) was the concentration of acetic minus propionic and butyric acid divided by the total amount of SCFA. The index reflects the fermentation of carbohydrates and the proinflammatory effect of SCFA. It was constructed as a balance between the proinflammatory effects of acetic acid and the anti-inflammatory effects of butyric and propionic acids [34].
- Index B (proteolytic fermentation) was the sum of i-butyric and i-valeric acids. The index reflects the fermentation of proteins and the anti-inflammatory effects of SCFA [34].

### 2.5. Statistics

**Figure 1:**
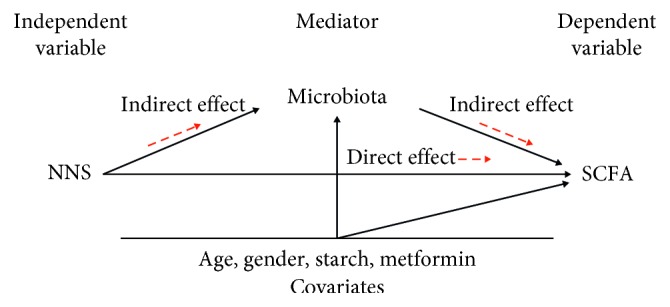
A directed acyclic graph of the direct and indirect effects (mediated via the microbiota) of nonnutritive sweeteners (NNSs) on short-chain fatty acids (SCFA) adjusted for age, gender, and intake of starch and metformin.

Descriptive statistics are given as number and proportion (%), mean with standard deviation (SD), or median with range. The mediation analyses were carried out as follows: first, linear regression analyses were used with each SCFA as the dependent variable, one at a time, and NNSs, starch, metformin, age, and gender as independent variables to identify SCFA with a statistically significant total effect of NNSs. Second, linear or ordinal logistic regression analyses were used with each of the 39 candidate microbiota and the dysbiosis indices, one at a time, as the dependent variable and the aforementioned independent variables, to identify the microbiota with statistically significant association with NNSs. Third, for the combinations of SCFA microbiota, mediation analyses were carried out as described by Hayes AF et al. [35, 36], to estimate direct and indirect effects (mediated through the bacterial groups at different taxonomic levels) of NNSs on SCFA, with age, gender, starch, and metformin as covariates. Bootstrap confidence intervals based on 5000 bootstrap replications was calculated for the indirect effect. The method does not allow the calculation of *p* values for the indirect effects.  Figure 1 shows a directed acyclic graph of the mediation model. *p* values <0.05 were judged as being statistically significant. To adjust for multiple testing, Benjamini–Hochberg false discovery rate-adjusted *q* values were calculated in *R* for the associations between NNSs and the individual SCFA and reported for *p* values below 0.05. Other analyses were performed with IBM SPSS Statistics for Windows, version 25.0 (IBM Corp., Armonk, NY, USA).

### 2.6. Ethical Approval

The study was approved by the Norwegian Regional Committees for Medical and Health Research Ethics (reference number 2012/966) and was performed in accordance with the Declaration of Helsinki. All the participants gave written informed consent before inclusion.

## 3. Results

### 3.1. Subject Characteristics

Eighty-nine out of 350 consecutive subjects with morbid obesity in the period from December 2012 to September 2014 were included in the study. Reasons for exclusions were as follows: study nurse was unavailable (no = 111), refused participation (no = 80), erroneously included (no = 17), and did not fill in the food frequency questionnaire or provide faecal samples (no = 53).  Table 1 gives the characteristics of the 89 participants.

### 3.2. Short-Chain Fatty Acids

**Figure 2:**
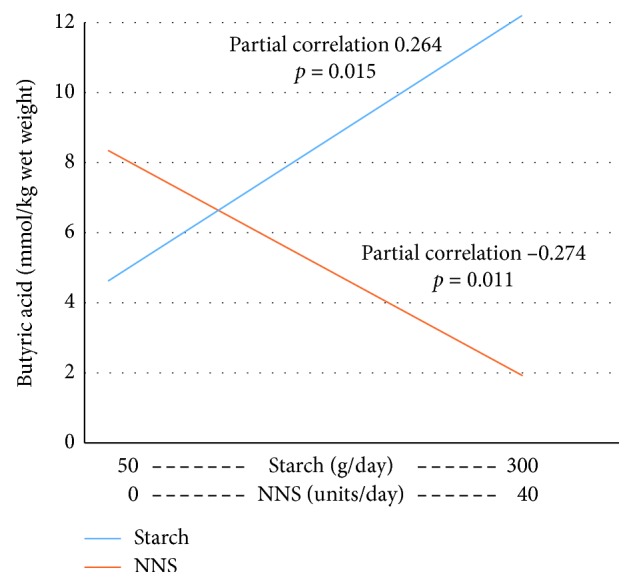
Regression lines for the associations between starch and NNSs (on the *x*-axis) and butyric acid (on the *y*-axis) calculated at the mean of the covariates age, gender, use of metformin, and/or NNSs/starch.

Table 2 gives the associations between SCFA and starch, NNSs, and metformin adjusted for age and gender. NNSs were negatively associated with butyric acid and valeric acid.  Figure 2 shows the associations between butyric acid and starch and NNSs adjusted for the means of age, gender, metformin, and/or NNSs/starch.

### 3.3. Faecal Microbiota

NNSs were positively associated with the DI and negatively with the ADI, and associated with four out of the 39 bacteria reported by the commercially available test.  Table 3 gives the statistically significant associations between NNSs and the faecal microbiota adjusted for starch, metformin, age, and gender.

### 3.4. Direct and Indirect Effects of NNSs on SCFA

NNSs were associated with butyric acid and valeric acid, and with the dysbiosis indices and four of the bacteria groups.  Table 4 gives the direct and indirect effects of NNSs on butyric acid and valeric acid. The effects are shown only for the bacteria associated with NNSs. No statistically significant indirectly mediated effects were seen.

## 4. Discussion

This study in subjects with morbid obesity showed significant effects of the diet and drugs on the faecal microbiota and SCFA. The most important finding was the association between the use of NNSs and reduced butyric acid. Similar effects of NNSs have been observed in mice [37].

### 4.1. Physiological Effects of Butyric Acid and NNSs

Butyric acid has multiple potential beneficial effects of particular importance for subjects with obesity [38]. Butyric acid reduces appetite, induces sustained satiety, promotes energy expenditure and fat oxidation by activation of brown adipose tissue, reduces insulin resistance, and improves dyslipidaemia [11, 12, 39, 40]. In mice, butyric acid prevents dietary-induced weight gain and induces significant weight loss [12, 39]. These effects are, without exception, favourable for subjects with obesity. The effects are in part mediated via gut-brain neural circuits [12, 41]. The discrepant sensory and metabolic signals (taste-calorie uncoupling) to the intake of NNSs modify the brain response to food and could have long-term consequences for food intake [42]. The adverse physiological effects associated with NNSs (such as metabolic changes with glucose intolerance, increased appetite and weight gain, weaker caloric compensation, and neurophysiological and brain dysfunction) are similar to those associated with butyric acid depletion and could in part be due to butyric acid reduction.

### 4.2. Clinical Effects of NNSs

The clinical usefulness of NNSs for weight control is uncertain. Intake of NNSs, often in large amounts, combined with an attempt to reduce intake of food rich in sugar are common in subjects with obesity aiming at weight reduction. In this study, intake of 20–43 units/day (corresponding to 2.0–4.3 litres of carbonated beverages with NNSs) was rather common. NNSs have been associated with an unhealthy lifestyle [27]. The clinically relevant favourable and unfavourable effects of NNSs in humans are still under debate despite comprehensive research. A recently published systematic review and meta-analyses concluded that no evidence of any effect of NSSs was seen on overweight and obese adults trying to lose weight, and that potential harms could not be excluded [43].

### 4.3. Comparisons of the Clinical and Physiological Effects of NNSs

The clinical and physiological effects of NNSs seem discordant. The favourable effects of a modest reduction of the intake of sugar by subjects using NNSs could be counteracted by the unfavourable physiological effects. The clinical relevance and physiological effects of reduced valeric acid associated with NNSs are unknown.

### 4.4. The Diet and the Faecal Microbiota

The faecal microbiota and its metabolites are highly influenced by the diet [5, 19]. NNSs were associated with an unfavourable dysbiosis, reduced amounts of *Faecalibacterium prausnitzii* and *Bacteroides fragilis*, and increased amounts of *Ruminococcus gnavus* and *Streptococcus* spp. Changes in the gut microbiota are common in subjects with obesity, and an increased Firmicutes/Bacteroidetes ratio has often been reported [6, 16, 17]. Increased *Ruminococcus gnavus* and *Streptococcus* spp. that are part of the Firmicutes and reduced *Bacteroides fragilis* that is part of the Bacteroidetes could indicate an increase in Firmicutes/Bacteroidetes ratio. *Faecalibacterium prausnitzii* has been ascribed important health-related effect also in subjects with obesity [14, 44, 45].

### 4.5. The Diet and Drugs and Butyric Acid

The positive association between intake of starch and faecal butyric acid was expected. The microbes use resistant nondigestible carbohydrates and fibre such as slowly digestible and resistant starch for production of SCFA, in particular butyric and propionic acids [19, 46, 47]. Low-carbohydrate diets such as a low content of rapidly digestible starch reduce faecal butyric acid [19]. The antiobesity effect of slowly digestible and resistant starch, which is due to increased energy expenditure and not reduced caloric intake, is likely mediated by increased microbial butyric acid production [48]. The intake of fibre and starch was significantly correlated, and associations between fibre and SCFA were expected but not found. Therefore, starch was used in the analyses. No associations were seen between metformin and the SCFA. Since metformin was used by nearly all subjects with diabetes, the effects of diabetes and metformin are difficult to separate. Metformin was used in this study because other studies have shown that metformin and not diabetes is the main contributor to the microbial alterations [21, 49].

### 4.6. The Microbiota and SCFA

Several microbes, of which *Faecalibacterium prausnitzii* has been mentioned as the most important one, have the capability for production of butyric acid by different metabolic pathways [45, 47]. Four groups of microbes including *Faecalibacterium prausnitzii* were associated with intake of NNSs. It was unexpected that neither *Faecalibacterium prausnitzii* nor any of the other bacterial groups were significant mediators of the negative association between NNSs and butyric acid. Since a direct effect of NNSs on butyric acid is unlikely, bacterial groups not specified by the commercially available microbial test probably mediated the effect.

### 4.7. Other Effects of the Microbiota and SCFA

In a previous study with the same methods, we reported the associations between the microbiota and SCFA and psychobiological comorbidity [28]. In contrast to this study with clear and significant associations between dietary factors and butyric acid with possible clinical consequences, the previous study showed a high number of significant and partly divergent associations and revealed no straightforward gut-brain communication pathways.

### 4.8. Strengths and Limitations

The participants with morbid obesity in need of weight reduction and therefore having a high intake of NNSs and referred for evaluation of bariatric surgery were well suited for the study of health-related effects of NNSs. The external validity is, however, restricted to this group. Measurement of SCFA in faeces and not in proximal parts of colon might be a limitation. Faecal concentration of SCFA is a poor estimate of colonic SCFA production, but the study did not aim to quantify colonic SCFA production. However, SCFA present in faeces is highly dependent on colonic SCFA production. The microbial test measured only the amount of 39 bacterial groups at different taxonomic levels relative to a reference population and on a coarse scale. A precise measurement of more bacterial groups could have improved the results. The effects might differ between various NNSs, and information about types of NNSs was not available. The results are based on a high number of statistical tests, mostly correlations, and type I errors cannot be excluded. Adjustment for multiple testing was performed for the main findings, which were the associations between NNSs and the individual SCFA. Multiplicity adjustment is a field of much research and controversy. The influential epidemiologist Kenneth Rothman argues against multiplicity adjustment in many settings [50]. In this study, adjustment for multiple testing was added to the other analyses for the most important findings. The analyses, which give very conservative results, showed a clear trend for an association between NNSs and butyric acid and strengthened the findings. The presence of unknown confounders cannot be excluded.

## 5. Conclusions

Faecal butyric acid was positively and negatively associated with the use of starch, which has been claimed to have antiobesogenic effects, and NNSs, respectively. The measured bacterial groups did not mediate these effects. Lack of butyric acid has weight-inducing effects and metabolic consequences that are unfavourable for subjects with obesity. The negative association between NNSs and butyric acid could indicate an obesogenic effect of NNSs. Since there is no evidence for a weight-reducing effect of NNSs on subjects with obesity and NNSs might counteract the favourable effects of butyric acid, it seems imprudent to use NNSs for weight reduction.

## Figures and Tables

**Table 1:** Characteristics of the 89 subjects (the number of patients is given in brackets if less than 89).

Subject characteristics	Number (%) (mean and/or median)	SD and/or range
Gender (male/female)	14 (15.7%)/75 (84.3)	
Age (years)	44.6	8.7
Height (cm)	170	8.1
Weight (kg)	121.1	16.4
BMI (kg/m^2^)	41.8	3.6
Coffee (cups/day)	3.0	2.3
Smoking (daily/previously/never)	11 (12.4%)/42 (47.2%)/36 (40.4%)	
Diabetes (no 86) (yes/no)	20 (23.3%)/66 (76.7%)	
Metformin use (yes/no)	16 (18.0%)/73 (82.0%)	
Protein (g/day)	110 (median 106)	36 (40 to 213)
Fat (g/day)	97 (median 87)	47 (21 to 283)
Carbohydrates (g/day)	273 (median 246)	128 (65 to 903)
Sugar (g/day)	45 (median 25)	75 (0.8 to 632)
Starch (g/day)	132 (median 122)	51 (24 to 336)
Fibre (g/day)	35 (median 35)	11 (12 to 72)
Nonnutritive sweeteners (units^*∗*^/day)	7.5 (median 3.2)	10 (0 to 43)
Dysbiosis (yes/no)	58 (65%)/31 (35%)	
Dysbiosis Index (score 1–5)	3.0 (median 3.0)	1.3 (1 to 5)
Alternative dysbiosis index (−14–14)	−0.5 (median 0.0)	2.7 (−8.0 to 7.0)
SCFA total (mmol/kg wet weight)	36.7 (median 28.7)	21.6 (5.9 to 149.2)
Acetic acid (mmol/kg wet weight)	19.9 (median 16.4)	10.8 (2.9 to 67.9)
Propionic acid (mmol/kg wet weight)	6.4 (median 5.2)	4.2 (1.3 to 25.6)
Isobutyric acid (mmol/kg wet weight)	0.7 (median 0.7)	0.6 (0.0 to 5.2)
Butyric acid (mmol/kg wet weight)	7.2 (median 5.6)	5.4 (1.0 to 34.5)
Isovaleric acid (mmol/kg wet weight)	1.1 (median 0.9)	1.0 (0.0 to 7.8)
Valeric acid (mmol/kg wet weight)	1.0 (median 0.8)	0.9 (0.0 to 5.1)
Isocapronic acid (mmol/kg wet weight)	0.0 (median 0.0)	0.0 (0.00 to 0.08)
Capronic acid (mmol/kg wet weight)	0.32 (median 0.06)	0.53 (0.00 to 3.10)
Index A^†^	0.19 (median 0.19)	0.10 (−0.11 to 0.44)
Index B^#^	1.84 (median 1.55)	1.61 (0.00 to 13.04)

^*∗*^One unit = 100 mL beverage with nonnutritive sweeteners or 2 tablets/teaspoons of nonnutritive sweeteners for coffee or tea. ^†^Index A: saccharolytic fermentation, i.e., the concentration of acetic minus propionic and butyric acid divided by the total amount of SCFA. ^#^Index B: proteolytic fermentation, i.e., the sum of concentrations of i-butyric and i-valeric acid.

**Table 2:** Linear regression analyses with the SCFA one at a time as the dependent variable and starch, nonnutritive sweeteners, metformin, gender (not shown), and age (not shown) as simultaneous independent variables.

Dependent variable	Starch^#^			Nonnutritive sweeteners			Metformin		
	B (95% CI)	*p* value	Partial corr.	B (95% CI)	*p* value	Partial corr.	B (95% CI)	*p* value	Partial corr.
Total SCFA	0.108 (0.009, 0.206)	0.032	0.232	−0.488 (−0.986, 0.009)	0.054	−0.210	11.1 (1.9, 24.0)	0.09	0.184
Acetic acid	0.057 (0.008, 0.106)	0.024	0.244	−0.207 (−0.455, 0.042)	0.102	−0.179	5.3 (−1.1, 11.8)	0.106	0.177
Propionic acid	0.017 (−0.002, 0.036)	0.084	0.189	−0.066 (−0.164, 0.032)	0.183	−0.146	2.1 (−0.4, 4.7)	0.104	0.178
Isobutyric acid	0.001 (−0.002, 0.004)	0.503	0.074	−0.011 (−0.026, 0.004)	0.139	−0.162	0.4 (0.0, 0.8)	0.045	0.218
Butyric acid	0.030 (0.006, 0.054)	0.015	0.264	−0.159 (−0.280, −0.037)	0.011^*∗*^	−0.274	2.1 (−1.1, 5.2)	0.200	0.140
Isovaleric acid	0.001 (−0.003, 0.006)	0.529	0.069	−0.016 (−0.038, 0.007)	0.170	−0.150	0.6 (0.0, 1.2)	0.046	0.217
Valeric acid	0.001 (−0.003, 0.005)	0.603	0.057	−0.022 (−0.043, −0.002)	0.029^*∗*^	−0.237	0.5 (−0.02, 1.0)	0.061	0.204
Isocapronic acid	0.000 (0.000, 0.000)	0.746	0.036	0.000 (0.000, 0.000)	0.438	0.085	0.01 (0.00, 0.02)	0.002	0.327
Capronic acid	0.000 (−0.002, 0.003)	0.819	0.025	−0.008 (−0.020, 0.004)	0.205	−0.139	0.11 (−0.22, 0.43)	0.513	0.072
Index A	0.000 (−0.001, 0.000)	0.705	−0.042	0.001 (−0.001, 0.004)	0.251	0.126	0.005 (−0.06, 0.07)	0.873	0.018
Index B	0.002 (−0.005, 0.010)	0.517	0.071	−0.027 (−0.063, 0.010)	0.156	−0.155	0.98 (0.02, 1.95)	0.045	0.218

^*∗*^False discovery rate-adjusted *q* values for butyric and valeric acids were 0.088 and 0.116, respectively. ^**#**^Fibre and starch were significantly correlated (Pearson's *r* = 0.60, *p* < 0.001). Since fibre was not significantly associated with either butyric acid or valeric acid, starch was used in the analyses.

**Table 3:** Linear and ordinal regression analyses with the dysbiosis indices one at a time, and the six out of 41 faecal bacterial species/groups that were statistically significantly associated with nonnutritive sweeteners as the dependent variable, and starch, nonnutritive sweeteners, metformin, age (not shown), and gender (not shown) as independent variables.

Dependent variable	Starch		Nonnutritive sweeteners		Metformin	
	B (95% CI)	*p* value	B (95% CI)	*p* value	B (95% CI)	*p* value
*Dysbiosis Index* ^*∗*^	−0.002 (−0.007, 0.003)	0.455	0.049 (0.022, 0.077)	0.001	0.834 (0.122, 1.546)	0.022
*Alternative Dysbiosis Index* ^*∗*^	−0.007 (−0.018, 0.004)	0.198	−0.090 (−0.143, −0.036)	0.001	1.676 (0.279, 3.074)	0.019
*Faecalibacterium prausnitzii* ^†^	−0.003 (−0.012, 0.007)	0.583	−0.056 (−0.103, −0.009)	0.019	−1.142 (−2.312, 0.028)	0.056
*Bacteroides fragilis* ^†^	−0.003 (−0.013, 0.008)	0.610	0.074 (0.025, 0.122)	0.003	0.416 (−0.899, 1.730)	0.536
*Ruminococcus gnavus* ^†^	0.005 (−0.009, 0.018)	0.520	0.069 (0.009, 0.128)	0.024	0.793 (−0.928, 2.514)	0.367
*Streptococcus* spp.^†^	−0.009 (−0.023, 0.004)	0.156	0.093 (0.036, 0.150)	0.001	−0.658 (−2.143, 0.826)	0.385

^*∗*^Linear regression analyses. ^†^Ordinal regression analyses.

**Table 4:** The total, direct, and indirect (mediated) effects of NNSs on faecal SCFA. The results are presented for the SCFA that were statistically significantly associated with NNSs, and the indirect (mediated) effects are shown for the bacterial groups and species significantly associated with NNSs.

Dependent variable	Mediator	Total effect of NNS B (95% CI), *p* value	Direct effect of NNS B (95% CI), *p* value	Indirect effect via mediator B (95% CI)
Butyric acid	Dysbiosis Index	−0.159 (−0.281 to −0.037), 0.011	−0.148 (−0.279 to −0.016), 0.029	−0.011 (−0.061 to 0.033)
Butyric acid	Alternative Dysbiosis Index	−0.159 (−0.281 to −0.037), 0.011	−0.159 (−0.289 to −0.029), 0.016	0.000 (−0.040 to 0.049)
Butyric acid	*Faecalibacterium prausnitzii*	−0.159 (−0.281 to −0.037), 0.011	−0.176 (−0.303 to −0.049), 0.007	0.017 (−0.022 to 0.073)
Butyric acid	*Bacteroides fragilis*	−0.159 (−0.281 to −0.037), 0.011	−0.149 (−0.281 to −0.017), 0.027	−0.010 (−0.051 to 0.024)
Butyric acid	*Ruminococcus gnavus*	−0.159 (−0.281 to −0.037), 0.011	−0.162 (−0.292 to −0.031), 0.016	0.003 (−0.023 to 0.048)
Butyric acid	*Streptococcus* spp.	−0.159 (−0.281 to −0.037), 0.011	−0.152 (−0.282 to −0.023), 0.022	−0.006 (−0.034 to 0.018)
Valeric acid	Dysbiosis Index	−0.022 (−0.043 to −0.002), 0.029	−0.025 (−0.046 to −0.003), 0.027	0.002 (−0.004 to 0.010)
Valeric acid	Alternative Dysbiosis Index	−0.022 (−0.043 to −0.002), 0.029	−0.022 (−0.044 to −0.001), 0.045	−0.001 (−0.008 to 0.008)
Valeric acid	*Faecalibacterium prausnitzii*	−0.022 (−0.043 to −0.002), 0.029	−0.029 (−0.050 to −0.009), 0.006	0.007 (−0.001 to 0.018)
Valeric acid	*Bacteroides fragilis*	−0.022 (−0.043 to −0.002), 0.029	−0.022 (−0.044 to −0.001), 0.046	−0.000 (−0.008 to 0.008)
Valeric acid	*Ruminococcus gnavus*	−0.022 (−0.043 to −0.002), 0.029	−0.020 (−0.041 to 0.002), 0.069	−0.003 (−0.010 to 0.006)
Valeric acid	*Streptococcus* spp.	−0.022 (−0.043 to −0.002), 0.029	−0.023 (−0.045 to −0.002), 0.032	−0.001 (−0.003 to 0.007)

## Data Availability

Case report forms (CRFs) on paper were used for collection of the clinical data, and all the CRFs are safely stored. The data were transferred manually to SPSS for statistical analyses. The data files are stored by Innlandet Hospital Trust, Brumunddal, Norway, on a server dedicated to research and with security according to the rules given by the Norwegian Data Protection Authority, P.O. Box 8177 Dep, NO-0034 Oslo, Norway. The data are available on request to the authors.

## References

[1] WHO (2019). *Fact Sheet N 311, Obesity and Overweight*.

[2] Abdelaal M., le Roux C. W., Docherty N. G. (2017). Morbidity and mortality associated with obesity. *Annals of Translational Medicine*.

[3] Guh D. P., Zhang W., Bansback N., Amarsi Z., Birmingham C. L., Anis A. H. (2009). The incidence of co-morbidities related to obesity and overweight: a systematic review and meta-analysis. *BMC Public Health*.

[4] WHO (2019). *Controlling the Global Obesity Epidemic*.

[5] David L. A., Maurice C. F., Carmody R. N. (2014). Diet rapidly and reproducibly alters the human gut microbiome. *Nature*.

[6] Bouter K. E., van Raalte D. H., Groen A. K., Nieuwdorp M. (2017). Role of the gut microbiome in the pathogenesis of obesity and obesity-related metabolic dysfunction. *Gastroenterology*.

[7] Del Chierico F., Abbatini F., Russo A. (2018). Gut microbiota markers in obese adolescent and adult patients: age-dependent differential patterns. *Frontiers in Microbiology*.

[8] Castaner O., Goday A., Park Y.-M. (2018). The gut microbiome profile in obesity: a systematic review. *International Journal of Endocrinology*.

[9] Gao R., Zhu C., Li H. (2018). Dysbiosis signatures of gut microbiota along the sequence from healthy, young patients to those with overweight and obesity. *Obesity*.

[10] Arora T., Sharma R., Frost G. (2011). Propionate. Anti-obesity and satiety enhancing factor?. *Appetite*.

[11] Chakraborti C. K. (2015). New-found link between microbiota and obesity. *World Journal of Gastrointestinal Pathophysiology*.

[12] Li Z., Yi C.-X., Katiraei S. (2018). Butyrate reduces appetite and activates brown adipose tissue via the gut-brain neural circuit. *Gut*.

[13] Al-Lahham S. A. H., Peppelenbosch M. P., Roelofsen H., Vonk R. J., Venema K. (2010). Biological effects of propionic acid in humans; metabolism, potential applications and underlying mechanisms. *Biochimica et Biophysica Acta (BBA)—Molecular and Cell Biology of Lipids*.

[14] Andoh A., Nishida A., Takahashi K. (2016). Comparison of the gut microbial community between obese and lean peoples using 16S gene sequencing in a Japanese population. *Journal of Clinical Biochemistry and Nutrition*.

[15] Koliada A., Syzenko G., Moseiko V. (2017). Association between body mass index and Firmicutes/Bacteroidetes ratio in an adult Ukrainian population. *BMC Microbiology*.

[16] Rajilic-Stojanovic M., de Vos W. M. (2014). The first 1000 cultured species of the human gastrointestinal microbiota. *FEMS Microbiology Reviews*.

[17] Bell D. S. H. (2015). Changes seen in gut bacteria content and distribution with obesity: causation or association?. *Postgraduate Medicine*.

[18] Wu G. D., Chen J., Hoffmann C. (2011). Linking long-term dietary patterns with gut microbial enterotypes. *Science*.

[19] Scott K. P., Duncan S. H., Flint H. J. (2008). Dietary fibre and the gut microbiota. *Nutrition Bulletin*.

[20] Montandon S. A., Jornayvaz F. R. (2017). Effects of antidiabetic drugs on gut microbiota composition. *Genes*.

[21] Wu H., Esteve E., Tremaroli V. (2017). Metformin alters the gut microbiome of individuals with treatment-naive type 2 diabetes, contributing to the therapeutic effects of the drug. *Nature Medicine*.

[22] Suez J., Korem T., Zeevi D. (2014). Artificial sweeteners induce glucose intolerance by altering the gut microbiota. *Nature*.

[23] Suez J., Korem T., Zilberman-Schapira G., Segal E., Elinav E. (2015). Non-caloric artificial sweeteners and the microbiome: findings and challenges. *Gut Microbes*.

[24] Feehley T., Nagler C. R. (2014). The weighty costs of non-caloric sweeteners. *Nature*.

[25] Farup P. G., Aasbrenn M., Valeur J. (2018). Separating “good” from “bad” faecal dysbiosis—evidence from two cross-sectional studies. *BMC Obesity*.

[26] Shanahan F., van Sinderen D., O’Toole P. W., Stanton C. (2017). Feeding the microbiota: transducer of nutrient signals for the host. *Gut*.

[27] Winther R., Aasbrenn M., Farup P. G. (2017). Intake of non-nutritive sweeteners is associated with an unhealthy lifestyle: a cross-sectional study in subjects with morbid obesity. *BMC Obesity*.

[28] Farup P., Valeur J. (2018). Faecal microbial markers and psychobiological disorders in subjects with morbid obesity. A cross-sectional study. *Behavioral Sciences*.

[29] The Norwegian food composition table, 2019, http://www.matvaretabellen.no/?language=en

[30] Casén C., Vebø H. C., Sekelja M. (2015). Deviations in human gut microbiota: a novel diagnostic test for determining dysbiosis in patients with IBS or IBD. *Alimentary Pharmacology & Therapeutics*.

[31] Genetic Analysis AS (2019). GAMap TM dysbiosis test. http://www.genetic-analysis.com/patent.

[32] Zijlstra J. B., Beukema J., Wolthers B. G., Byrne B. M., Groen A., Ankert J. D. (1977). Pretreatment methods prior to gaschromatographic analysis of volatile fatty acids from faecal samples. *Clinica Chimica Acta*.

[33] Høverstad T., Bjørneklett A., Midtvedt T., Fausa O., Bøhmer T. (1984). Short-chain fatty acids in the proximal gastrointestinal tract of healthy subjects. *Scandinavian Journal of Gastroenterology*.

[34] Tjellström B., Högberg L., Stenhammar L. (2012). Effect of exclusive enteral nutrition on gut microflora function in children with Crohn’s disease. *Scandinavian Journal of Gastroenterology*.

[35] Hayes A. F., Rockwood N. J. (2017). Regression-based statistical mediation and moderation analysis in clinical research: observations, recommendations, and implementation. *Behaviour Research and Therapy*.

[36] Hayes A. F. (2018). *Introduction to Mediation, Moderation, and Conditional Process Analysis: A Regression-Based Approach*.

[37] Uebanso T., Ohnishi A., Kitayama R. (2017). Effects of low-dose non-caloric sweetener consumption on gut microbiota in mice. *Nutrients*.

[38] Canani R. B., Costanzo M. D., Leone L., Pedata M., Meli R., Calignano A. (2011). Potential beneficial effects of butyrate in intestinal and extraintestinal diseases. *World Journal of Gastroenterology*.

[39] Whitt J., Woo V., Lee P. (2018). Disruption of epithelial HDAC3 in intestine prevents diet-induced obesity in mice. *Gastroenterology*.

[40] Fluitman K. S., Wijdeveld M., Nieuwdorp M., Ijzerman R. G. (2018). Potential of butyrate to influence food intake in mice and men. *Gut*.

[41] De Vadder F., Kovatcheva-Datchary P., Goncalves D. (2014). Microbiota-generated metabolites promote metabolic benefits via gut-brain neural circuits. *Cell*.

[42] Crézé C., Candal L., Cros J. (2018). The impact of caloric and non-caloric sweeteners on food intake and brain responses to food: a randomized crossover controlled trial in healthy humans. *Nutrients*.

[43] Toews I., Lohner S., Küllenberg de Gaudry D., Sommer H., Meerpohl J. J. (2019). Association between intake of non-sugar sweeteners and health outcomes: systematic review and meta-analyses of randomised and non-randomised controlled trials and observational studies. *BMJ*.

[44] Feng J., Tang H., Li M. (2014). The abundance of fecal *Faecalibacterium prausnitzii* in relation to obesity and gender in Chinese adults. *Archives of Microbiology*.

[45] Ferreira-Halder C. V., Faria A. V. d. S., Andrade S. S. (2017). Action and function of *Faecalibacterium prausnitzii* in health and disease. *Best Practice & Research Clinical Gastroenterology*.

[46] Flint H. J., Scott K. P., Duncan S. H., Louis P., Forano E. (2012). Microbial degradation of complex carbohydrates in the gut. *Gut Microbes*.

[47] Louis P., Flint H. J. (2017). Formation of propionate and butyrate by the human colonic microbiota. *Environmental Microbiology*.

[48] Luo K., Wang X., Zhang G. (2018). The anti-obesity effect of starch in a whole grain-like structural form. *Food & Function*.

[49] Forslund K., Hildebrand F., Nielsen T. (2015). Disentangling type 2 diabetes and metformin treatment signatures in the human gut microbiota. *Nature*.

[50] Rothman K. J. (2014). Six persistent research misconceptions. *Journal of General Internal Medicine*.

